# Comparison of Four Dietary Pattern Indices in Australian Baby Boomers: Findings from the Busselton Healthy Ageing Study

**DOI:** 10.3390/nu15030659

**Published:** 2023-01-28

**Authors:** Sierra R. McDowell, Kevin Murray, Michael Hunter, Lauren C. Blekkenhorst, Joshua R. Lewis, Jonathan M. Hodgson, Nicola P. Bondonno

**Affiliations:** 1 School of Population and Global Health, University of Western Australia, Perth, WA 6009, Australia; 2 Busselton Population and Medical Research Institute, Busselton, WA 6280, Australia; 3 Nutrition & Health Innovation Research Institute, School of Medical and Health Sciences, Edith Cowan University, Perth, WA 6027, Australia; 4 Medical School, University of Western Australia, Perth, WA 6009, Australia; 5 Centre for Kidney Research, Children’s Hospital at Westmead, School of Public Health, Sydney Medical School, The University of Sydney, Sydney, NSW 2006, Australia; 6 The Danish Cancer Society Research Centre, 2100 Copenhagen, Denmark

**Keywords:** diet quality, dietary patterns, dietary guideline index 2013, EAT-lancet, Mediterranean diet

## Abstract

The assessment of dietary patterns comprehensively represents the totality of the diet, an important risk factor for many chronic diseases. This study aimed to characterise and compare four dietary pattern indices in middle-aged Australian adults. In 3458 participants (55% female) from the Busselton Healthy Ageing Study (Phase Two), a validated food frequency questionnaire was used to capture dietary data between 2016 and 2022. Four dietary patterns [Australian Dietary Guideline Index 2013 (DGI-2013); the Mediterranean Diet Index (MedDiet); the Literature-based Mediterranean Diet Index (Lit-MedDiet); and the EAT-Lancet Index], were calculated and compared by measuring total and sub-component scores, and concordance (𝜌_c_). Cross-sectional associations between the dietary indices and demographic, lifestyle, and medical conditions were modelled with linear regression and restricted cubic splines. Participants had the highest standardised scores for the DGI-2013 followed by the EAT-Lancet Index and the MedDiet, with the lowest standardised scores observed for the Lit-MedDiet. The DGI-2013 had the lowest agreement with the other scores (𝜌_c_ ≤ 0.47). These findings indicate that the diets included in this Australian cohort align more closely with the Australian Dietary Guidelines than with the other international dietary patterns, likely due to the wide variation of individual food group weightings in the construction of these indices.

## 1. Introduction

Diet is a major contributor to the risk of chronic disease, both in Australia and globally [1]. While the burden of disease attributed to diet has declined in Australia since 1990 [1], dietary risk factors still contribute to a relatively high proportion of the non-communicable disease burden, particularly in adults aged 45–64 years [1]. In 2015, diet was one of the major risk factors for non-communicable disease deaths in Australia, contributing to 19.7% of these deaths that year [1]. Due to the significant burden of disease that is associated with a low-quality diet, the Australian Dietary Guidelines (ADG) were created to inform the public of healthy eating behaviours to improve health and wellbeing, and they were last updated in 2013 [2].

Dietary patterns are described as the frequency and variety of food and beverages that are habitually consumed in an individual’s diet [3]. Nutritional epidemiological research has moved towards investigating dietary patterns in addition to specific foods, nutrients and phytochemicals. This is because dietary patterns are better at representing individuals’ real-life dietary habits and more holistically reflect the complex interactions between food intake and nutrients [4]. By investigating diet from a pattern perspective, the cumulative effects of food consumed together can be analysed [5].

One of the most common methods of characterising dietary patterns in epidemiological research is by using dietary quality indices, also known as dietary indices [4]. These measures assess the entire diet in terms of quality and, sometimes, variety. Dietary indices are often constructed from national or international dietary guidelines and reflect adherence to a particular dietary pattern [4]. Examples of such indices, which we examined in this study, include the Dietary Guidelines Index-2013 (DGI-2013), which provides insight into how well study participants adhered to the 2013 ADG [6]; the EAT-Lancet Index, which reflects how well the diet of the participants aligns with a universal reference diet taking into consideration eating for health from a sustainable food system [7]; the Mediterranean Diet Index (MedDiet) [8] because it measures adherence to the dietary pattern with the most evidence to date for beneficial associations with health outcomes [9]; and the Literature-Based Mediterranean Diet Index (Lit-MedDiet), developed as an improved method of examining adherence to the Mediterranean diet with greater transferability in response to fundamental shortcomings in the MedDiet construction, namely that it’s cut-off values are population specific [10].

Very few studies have compared other dietary patterns against the DGI-2013 head-to-head in the same study population. This is valuable to examine because it will provide a more comprehensive understanding of complex dietary data in the population, from both an Australian and international dietary perspective [11]. Understanding the construction of the dietary indices is also vitally important, as differences in their underlying composition can result in variation in their predictive power when used in longitudinal studies [12] and may not be suitable for use in a particular population. Additionally, exploring how these dietary indices differentially relate to demographic and lifestyle characteristics provides further insight into whether any particular characteristics are associated with adherence to a certain diet, and whether that varies across different dietary patterns. Therefore, the present study aims to characterise the dietary pattern in middle-aged Australian adults by comparing adherence and concordance of the DGI-2013, EAT-Lancet Index, MedDiet, and Lit-MedDiet indices. Additionally, we aimed to examine associations between these indices and demographic, lifestyle factors and self-reported medical conditions.

## 2. Materials and Methods

### 2.1. Study Population

**Figure 1:**
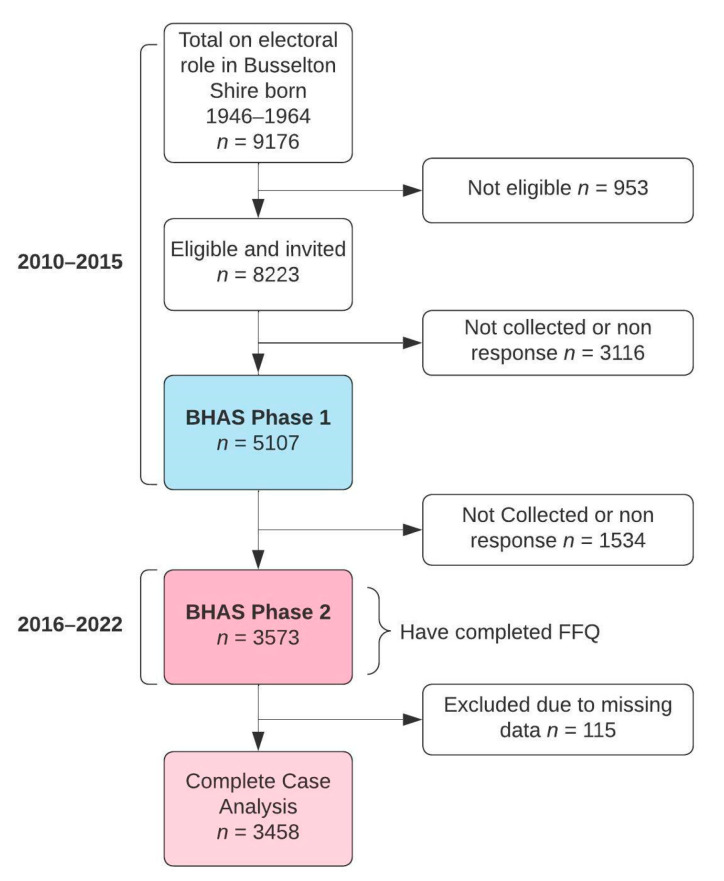
Participant flow chart.

The study sample is composed of participants from Phase Two of the Busselton Healthy Ageing Study (BHAS), a longitudinal study based on the recruitment of a sample of baby boomers (born between 1946 and 1964). BHAS Phase One was conducted between 2010 and 2015. All 8223 eligible (alive and non-institutionalised) adults listed on the electoral roll from the Busselton Shire in the South West Region of Western Australia were invited to participate in the study, with a total of 5107 participating in Phase One. The full protocol for the BHAS was described in detail previously [13]. The Phase Two data is the first phase follow-up of the BHAS and is the first phase to include extensive dietary data. The 5107 individuals who participated in Phase One of the BHAS were contacted again between 2016 and 2022 (~six years later) and were invited for a follow-up and to complete the comprehensive health questionnaire. As illustrated in Figure 1, out of the 5107 eligible individuals, 3573 participants took part in Phase Two and completed the questionnaire. Of these, 115 participants were excluded as they had missing data, leaving 3458 participants remaining for analysis in the present study.

### 2.2. Dietary Data

Participants completed a validated 145-item self-administered food frequency questionnaire (FFQ), with portion size and a nine-category frequency scale, ranging from “never” to “4+ servings per day”. The FFQ was previously assessed by the Blue Mountains Eye Study in a similarly aged population and was found to have reasonable validity, reliability and generalisability [14]. In the present study, participants reported their typical frequency of consumption of each food and beverage item over the previous 12 months. Using this data, four dietary indices were calculated as described below.

### 2.3. DGI-2013

The DGI-2013 includes 13 food categories that reflect the ADG, with each of the categories having a maximum score of 10, summing to give a maximum score of 130. A higher score reflects greater adherence to the ADG [6]. The specific details of the Index’s composition and cut-off values were described in detail previously [6] and have been adapted to this study. There were three key modifications in the calculation of the DGI-2013: 1. Alcohol was excluded from component eight as alcohol intake is already captured separately in component 13; 2. Component 12 considered only sugar added to foods by participants as foods high in added sugars are already considered in component eight (Limit intake of foods or drinks high in saturated fat, and containing added salt and sugar); 3. The calculation of component 1 was adjusted so that participants following a vegetarian diet would not be disproportionately penalised. The details of the DGI-2013 construction used in this study is provided in Table S1.

### 2.4. EAT-Lancet Index

A revised version of the EAT-Lancet Index with 14 food components and a graduated scoring system was used; the total score ranges from 0–42 points with a higher score indicating a higher adherence to the EAT-Lancet diet [15]. There were two key modifications in the calculation of the revised EAT-Lancet Index. 1. Beef/lamb/pork were combined into one category as many of the meat-related questions did not distinguish between the meat types. 2. Lard was included as a separate additional component, so its combination with unsaturated oil would total a maximum of three points; this addition provides a greater breadth of the relative fat intake, as it was originally intended [7]. The full breakdown of the revised EAT-Lancet construction used in this study is provided in Table S2.

### 2.5. MedDiet

The MedDiet is composed of nine food categories, each assigned either zero or one point based on median sex-specific intake: the total score ranges from 0–9 points [8]. The only category not to use median intake for its cut-point is alcohol, where grams per day of ethanol are used instead [8]. The median sex-specific intake of olive oil was used in this study instead of the saturated fat ratio as done previously [16]. The full breakdown of the MedDiet used in this study is provided in Table S3.

### 2.6. Lit-MedDiet

The Lit-MedDiet is composed of nine food categories, each assigned either zero, one or two points with points assigned based on intakes above or below predetermined cut-off values; the total score ranges from 0–18 points [10]. There was one modification in the calculation of the Lit-MedDiet; the cut-points for olive oil were changed from “Occasional use”, “Frequent use” and “Regular use”, to “Less than once per week”, “One to six times per week”, and “Daily/two or more times per day” respectively, to align with the frequency categories in the BHAS Phase Two FFQ. The full breakdown of the Lit-MedDiet used in this study is provided in Table S4.

The dietary indices for this paper were generated using SAS software version 9.4. Copyright © [2021] SAS Institute Inc. SAS and all other SAS Institute Inc. product or service names are registered trademarks or trademarks of SAS Institute Inc., Cary, NC, USA.

### 2.7. Covariates

Relevant variables captured in the BHAS Phase Two questionnaire as covariates were related to demographics, lifestyle and medical history. Demographic questions provided data on age (years), sex (male/female), marital status (partner/no partner), average annual total income before tax (≤$40,000; $40,001–$80,000; >$80,001; prefer not to say), education (high school; other educational institute; university), and ethnicity (Caucasian/other). Lifestyle factors included self-reported moderate to vigorous physical activity (the sum of (hours/week of moderate-intensity physical activities) + 2 × (hours/week of vigorous-intensity physical activities)) [17], and smoking status (never-smoker/former-smoker/current-smoker). Medical history questions provided data on self-reported medical conditions, both newly diagnosed in the past five years, and whether conditions previously reported in the baseline BHAS survey had persisted. Self-reported medical conditions included cardiovascular disease (defined as the self-reported incidence of angina/claudication/myocardial infarction (MIA)/ transient ischemic attack (TIA)/ stroke/carotid surgery/ coronary angiogram/coronary bypass), cancer, high blood pressure (ever and current), high cholesterol (ever and current), diabetes, kidney disease and chronic obstructive pulmonary disease (COPD).

### 2.8. Statistical Analysis

To describe adherence to each of the dietary patterns, the total score, and sub-components of each index, were presented for the study sample (BHAS Phase Two). The median (IQR) and percentage (*n*) of individuals who achieved each subcomponent score were calculated. Scores were also presented as percentages of the maximum possible score, and the mean [SD] of these percentages was calculated. Concordance between the four dietary indices (as z-scores) was measured using Lin’s concordance correlation coefficient (𝜌_c_), with 95% confidence intervals [18]. Additionally, density hexabin plots of z-scores were created to visually assess concordance between the dietary indices. Linear regression models were used to explore relationships between (i) demographics, (ii) lifestyle factors and (iii) self-reported medical conditions and the z-score responses of each of the four dietary scores [19]. Generally, in modelling, continuous variables were modelled using restricted cubic splines (knots placed at the 10th, 27.5th, 50th, 72.5th and 90th percentiles) [20], with likelihood ratio tests for non-linearity examined [21]. If the test for non-linearity was not significant, a linear term was fitted. The β coefficient estimates represented the change in the mean level of the dietary z-score to the reference level for categorical variables and the change in the mean dietary z-score for a one standard deviation increase around the mean of the explanatory variable of interest for continuous variables, estimated from restricted cubic spline modelling. Bootstrapping was used to obtain 95% confidence intervals for the effect of continuous variables. Three separate models were fitted for each dietary index response: 1. Adjustment for demographics only (age, sex, marital status, ethnicity, highest level of education attained, income); 2. Adjustment for demographics and lifestyle factors (all model one variables plus hours of vigorous to moderate physical exercise per week, and smoking status); 3. Adjustment for all the variables in model two plus self-reported medical conditions one at a time. Model three was replicated for each medical condition of interest. All statistical analyses were conducted using R version 4.1.2 [22].

## 3. Results

### 3.1. Baseline Characteristics

Of the 3458 participants included in the present study, 1912 (55%) were female, and the median (IQR) age was 64 (60–69) years. The baseline characteristics of the study population are presented in Table 1.

### 3.2. DGI-2013

**Figure 2:**
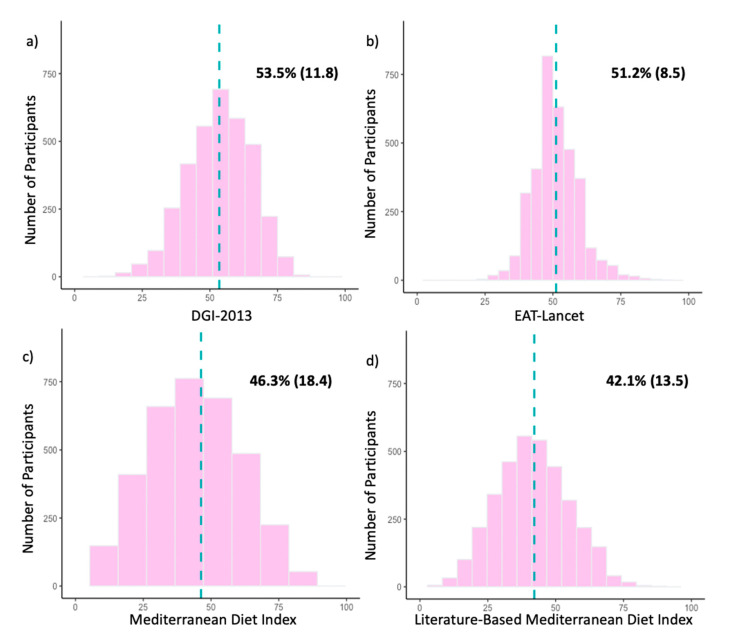
(**a**–**d**)**.** Histograms of participant scores for the (**a**) Dietary Guideline Index 2013 (DGI-2013), (**b**) EAT-Lancet Index, (**c**) Mediterranean Diet Index (MedDiet) and (**d**) Literature-Based Mediterranean Diet Index (Lit-MedDiet) for the whole study population (*n* = 3458). For comparison across indices, participant scores are represented as percentages of the maximum possible score for each dietary index. The dotted blue line represents the location of the mean. Mean percentages of participant scores are shown in the upper right-hand area of each histogram, presented as mean (SD).

Participant scores for the DGI-2013 ranged from 18–109 with a median (IQR) of 70.0 (59.0–80.5). The scores (when represented as a percentage of the total) were unimodal and symmetrically distributed with a mean [SD] of 53.5% [11.8] (Figure 2a). The subcomponents with the highest percentage of participants achieving a full score were meat (87.6%), alcohol (73.5%), fruit (66.9%) and added sugar (67.9%), whereas a low percentage of participants achieved a full score for variety (0%), lean meat ratio (3.4%), unsaturated fat (4.6%) and cereals (4.7%) (Table 2).

### 3.3. EAT-Lancet Index

For the EAT-Lancet Index, participant scores ranged from 9.5–39 with a median (IQR) of 21.5 (19.0–23.5). As seen in Figure 2b, the scores were unimodal and symmetrically distributed with a mean [SD] of 51.2% [8.5]. The highest percentage of participants achieving a full score was seen for lard (98.3%), poultry (85.9%) and vegetables (64.0%), whereas the lowest percentage was seen for unsaturated fat (0.2%), nuts (1.4%) and wholegrains (1.8%) (Table 2).

### 3.4. MedDiet

Participant scores for the MedDiet ranged from 0–9 with a median (IQR) of 4.0 (3.0–5.0). The scores (when represented as a percentage of the total) were unimodal and symmetrically distributed with a mean [SD] of 46.3% [18.4] (Figure 2c). The percentages of participants achieving a full subcomponent score were similar across the subcomponents, ranging from 46.2% to 1.4%, with the only outlier being olive oil (21.1%) (Table 2).

### 3.5. Lit-MedDiet

For the Lit-MedDiet, participant scores ranged from 1–16 with a median (IQR) of 8.0 (6.0–9.0). As seen in Figure 2d, the scores (when represented as a percentage of the total) were unimodal and symmetrically distributed with a mean [SD] of 42.1% [13.5]. The subcomponents with the highest percentage of participants achieving a full score were vegetables (85.7%) and fish (38.8%), whereas a low percentage of participants achieved a full score for olive oil (1.2%) and meat (17.6%) (Table 2).

### 3.6. Concordance between Dietary Indices

**Figure 3:**
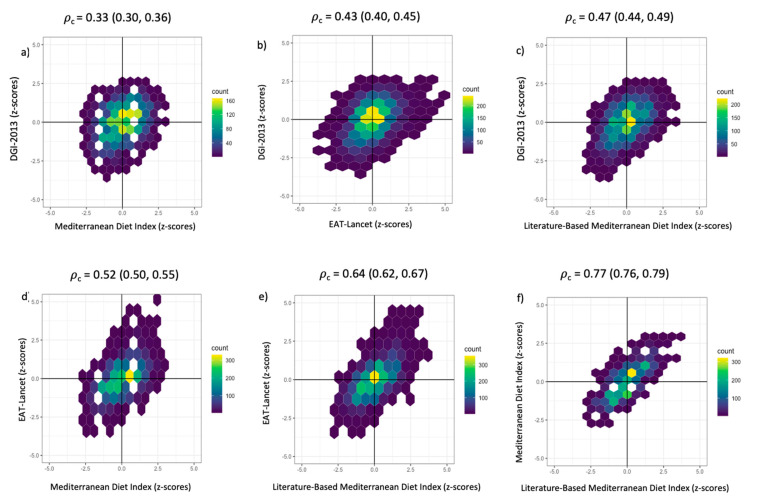
(**a**–**f**). Density hexabin plots with z-scores of the dietary indices: EAT-Lancet Index, Dietary Guideline Index 2013 (DGI-2013), Mediterranean Diet Index (MedDiet) and the Literature-Based Mediterranean Diet Index (Lit-MedDiet). Lin’s concordance correlation coefficient (𝜌_c_) and 95% confidence intervals were calculated to measure the extent of agreement pairwise between dietary indices.

The highest agreement was observed between the two MedDiet indices [𝜌_c_ = 0.77 (95% CI: 0.76, 0.79); Figure 3f], whereas the lowest agreement was observed between the DGI-2013 and the MedDiet indices [𝜌_c_ = 0.33 (95% CI: 0.30, 0.36); Figure 3a]. The DGI-2013 also had a low agreement with the other two dietary indices [𝜌_c_ < 0.47; Figure 3b,c]. This is also reflected in all density hexabin plots for the DGI-2013, with these plots having a higher proportion of participants in the top left and lower right quadrants than for the other three indices, indicating greater discordance (Figure 3).

### 3.7. Comparison of Demographic and Lifestyle Factors Associated with the Dietary Indices

After adjusting for other demographic and lifestyle factors, compared to males, females had higher DGI-2013, EAT-Lancet Index and Lit-MedDiet scores (Table 3). There was no significant difference in MedDiet between males and females, and while participants in the highest income bracket had a significantly higher MedDiet score [β 0.13 (95% CI, 0.03, 0.24)], there was no consistent pattern of those in higher income brackets having higher scores across all four dietary indices (Table 3). Compared to participants without a partner, those with a partner had significantly higher DGI-2013 scores [β 0.21 (95% CI, 0.13, 0.30)] (Table 3). Across all dietary indices, scores increased as education level increased. This pattern was consistent across all the dietary indices, with the highest observed in the EAT-Lancet Index [β 0.30 (95% CI, 0.21, 0.39)] (Table 3). Overall, the beta coefficients of the predictors for the MedDiet are generally lower compared to the Lit-MedDiet (Table 3).

**Figure 4:**
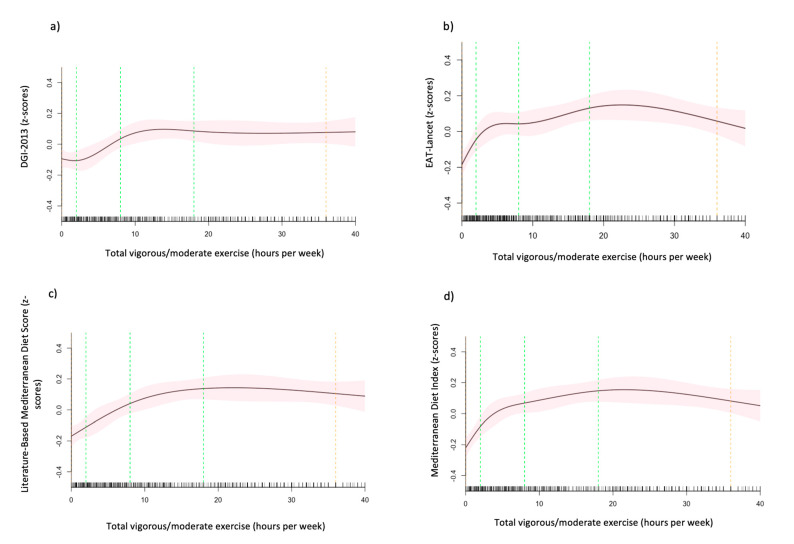
(**a**–**d**)**.** The association between hours of vigorous to moderate exercise per week and the (**a**) Dietary Guideline Index 2013 (DGI-2013), (**b**) EAT-Lancet Index, (**c**) Mediterranean Diet Index (MedDiet) and (**d**) Literature-Based Mediterranean Diet Index (Lit-MedDiet). Marginal mean estimates of the dietary indices (z-scores) are based on linear regression modelling, with hours of vigorous to moderate exercise per week fitted as a restricted cubic spline. Green dotted lines represent the location of the spline knots, and the orange dotted lines present the location of the spline bounds. Analyses are adjusted for age, sex, marital status, ethnicity, the highest level of education attained, income, hours of vigorous to moderate physical exercise per week and smoking status (model 2).

After adjusting for other demographic and lifestyle factors, compared to never-smokers, current-smokers had significantly lower DGI-2013 [β −0.48 (95% CI, −0.63, −0.33)], EAT-Lancet Index [β −0.41 (95% CI, −0.57, −0.25)] and Lit-MedDiet scores [β −0.24 (95% CI, −0.40, −0.08)], with the largest difference observed in the DGI-2013 (Table 3). The graphical representation of the non-linear association between hours of vigorous to moderate exercise per week and the four dietary scores (Figure 4) shows an initial positive association between total hours of vigorous to moderate exercise per week and the EAT-Lancet Index, MedDiet and the Lit-MedDiet. The initial slope for MedDiet and EAT-Lancet is steeper than the Lit-MedDiet. All the curves are a similar rounded shape, except for the DGI-2013 which has an initial dip, then a positive slope that plateaus after approximately 10 h per week (Figure 4a).

### 3.8. Comparison of Medical Factors Associated with the Dietary Indices

After adjusting for other demographic and lifestyle factors, those with diabetes (ever) had significantly higher DGI-2013 scores [β 0.16 (95% CI, 0.05, 0.28)] and lower EAT-Lancet Index [β −0.10 (95% CI,- 0.22, −0.02)], MedDiet [β −0.24 (95% CI, −0.36, −0.11)] and Lit-MedDiet scores [β −0.15 (95% CI, −0.28, −0.03)], than those without diabetes (Table 4). Participants with high blood pressure (ever) had significantly lower scores in EAT-Lancet Index [β −0.08 (95% CI, −0.15, −0.01)] and Lit-MedDiet [β −0.09 (95% CI, −0.17, −0.02)], and borderline significantly lower MedDiet scores [β −0.08 (95% CI, −0.15, 0.00)] (Table 4). Those with current high blood pressure had significantly lower EAT-Lancet Index [β −0.08 (95% CI, −0.18, −0.03)], MedDiet and Lit-MedDiet scores [β −0.10 (95% CI, −0.17, −0.02)] (Table 4).

## 4. Discussion

Out of the four dietary indices assessed in this cross-sectional data, participants scored highest for the DGI-2013 followed by the EAT-Lancet Index and the MedDiet, with the lowest scores observed for the Lit-MedDiet. Concordance between the dietary indices was generally low, with the DGI-2013 consistently having the lowest agreement with the other indices. The weighting of individual food components varied greatly across the four dietary indices. In general, females, participants with a partner and those with a higher level of education and exercise tended to score higher, whereas current smokers tended to score lower across the indices. This is consistent with previous studies that examined dietary quality with these indices individually [6,15,23,24,25]. However, our study is the first to observe this across all these indices in the same population.

Across the literature, few studies have described and compared more than one dietary index head-to-head in the same population, with even fewer drawing comparisons between any of the four indices calculated in the current study. In two studies of middle-aged Australian adults comparing the DGI-2013 and MedDiet indices [26,27], participants scored higher in the DGI-2013, as seen in the present study. It is perhaps unsurprising that the diets of these three Australian cohorts align more closely with the ADG than with a Mediterranean diet. However, given the wealth of evidence for the health benefits of a Mediterranean diet [9,28,29], the low concordance between the DGI-2013 and both the MedDiet and the Lit-MedDiet observed in the present study is noteworthy. As expected, the highest agreement was observed between the MedDiet and Lit-MedDiet scores, although a concordance of only 0.77 is surprising considering the two indices are measuring adherence to the same dietary pattern. Our study indicated that the Lit-MedDiet is a superior index to the MedDiet. One reason for this is that any sex-specific associations were likely negated due to the sex-specific median cut-points used in the MedDiet. This was seen previously where the mean DGI-2013 score was significantly higher for females than males, but with no significant difference for the MedDiet [26]. Furthermore, consistent with what other studies have highlighted [10,11,25], our findings suggest that, overall, the MedDiet is more of a crude measure compared to the Lit-MedDiet in a non-Mediterranean population. Our results highlighted that there are major differences in the underlying components between the DGI-2013 and the Lit-MedDiet, possibly contributing to the discordance between the indices. For example, the DGI-2013 positively scores a high intake of meat plus alternatives such as legumes, eggs etc., while the Lit-MedDiet positively scored for a low intake of meat. This likely accounts for the higher proportion of the cohort meeting the recommendations for meat intake in the DGI-2013, than in the Lit-MedDiet. Another difference was observed in the alcohol component, where participants scored well in the DGI-2013, but poorly in the Lit-MedDiet. This is likely due to the Lit-MedDiet positively scoring for moderate alcohol intake and the cohort having low alcohol intake, so they performed better when it was positively scored for low intake in the DGI-2013. The question remains whether the ADG needs to be modified to incorporate elements of the Mediterranean diet to align more closely with a Mediterranean diet. This could be achieved by enhancing the ADG to discriminate between protein types for a high intake of plant-based protein and a low intake of meat. It was suggested that translating a MedDiet in a non-Mediterranean population is possible, but flexibility is needed when adapting components to another country while retaining the fundamental parts of the Mediterranean diet [30]. However, the feasibility of adopting a Mediterranean diet in an Australian dietary context remains unclear and would require more research [31]. In the present study, the population adhered poorly to some of the essential components of the Mediterranean diet, such as high olive oil intake and low intake of red meat and dairy, suggesting that this population’s dietary patterns do not align with a Mediterranean diet.

Like the MedDiet, the EAT-Lancet diet is an international plant-based diet. Therefore, it is also not unexpected that our cohort’s dietary patterns also had closer alignment to the DGI-2013 than to the EAT-Lancet Index. Similar to the Lit-MedDiet, our results show variation in the cohort meeting individual food group recommendations between the DGI-2013 and the EAT-Lancet Index. For example, the cohort scored much better in the DGI-2013 compared to the EAT-Lancet Index for the wholegrain component. This difference is likely attributed to the DGI-2013 using a wholegrain-to-white bread ratio as a measure of relative wholegrain intake, whereas the EAT-Lancet Index uses the total intake of all whole grains, suggesting that in its current form, the DGI-2013 is not capturing the true picture for wholegrain intake. In this instance, this is more of a limitation in how the DGI-2013 is capturing wholegrain intake, rather than ADG recommendations, and the method used in the EAT-Lancet Index may be worth being considered for use in the DGI-2013 in future research, to discriminate wholegrain intake more accurately. Another difference was observed in the saturated fat component where the cohort performed better in the EAT-Lancet Index, likely due to lard not being commonly consumed in a typical Australian diet; lard is, therefore, not the ideal measure of saturated fat in an Australian context. A recent Australian study also found there are considerable differences between the ADGs and the EAT-Lancet dietary pattern, largely attributed to the EAT-Lancet diet recommendations of meat intake and discretionary foods being lower than in the ADGs [32]. Given the environmental benefits of the EAT-Lancet diet [33], the differences and low concordance observed in the present study between the DGI-2013 and the EAT-Lancet Index highlight the need for further research to evaluate the potential benefits of incorporating elements of these plant-based dietary patterns into the ADG, which is currently undergoing revisions with updated guidelines estimated to be released in 2024 [34]. Other European countries such as the UK [35], the Netherlands [36] and Sweden and Germany have started to incorporate the EAT-Lancet diet or similar dietary recommendations into their dietary guidelines [37]. Additionally, there has been research into incorporating both the EAT-Lancet and Mediterranean diet recommendations into the Italian national dietary guidelines [38]. However, as with the Mediterranean diet, adopting the EAT-Lancet diet recommendations would need to be tailored to an Australian context, as not all components may align with what is commonly eaten. While studies examining other Australian populations have found that lower socioeconomic status is associated with lower adherence to the ADG [39,40], in the present study there was no consistent effect of income on adherence to a particular dietary pattern in the population. However, this was not unexpected given that our population consisted of older adults in retirement age, where income would not necessarily be representative of economic status. Further research investigating the socioeconomic status and dietary patterns by comparing associations between the DGI-2013 to the EAT-Lancet indices would be valuable, given that it has been suggested that following the EAT-Lancet diet recommendations would be less expensive than the ADG [41]. Another factor to consider is that due to the small numbers of non-Caucasian participants in the population, we could not make any meaningful interpretations from any associations with ethnicity. While we expected an Australian population to have the strongest adherence to the ADG, it is likely that adherence would be different in other populations in Australia with more ethnic diversity, as seen previously [42].

### Strengths and Limitations

The results of the present study should be interpreted with the following limitations in mind: while it appears that our cohort scored the highest in the DGI-2013, we can’t draw conclusions about which index is overall a better measure of dietary quality because of their different underlying constructions. Only longitudinal studies will be able to evaluate their performance as predictors of health outcomes. When examining associations, we cannot assume causality nor rule out residual confounding by unmeasured factors. As per the nature of self-reported dietary assessment tools, FFQs can be prone to measurement error due to, but not limited to recall bias, lack of contextual information and details on food preparation and seasonal variation in the diet [14]. Additionally, while the FFQ used in this study was not designed to assess any specific dietary pattern [14], it was developed within an Australian dietary context, and therefore we cannot rule out an underlying bias towards the DGI-2013. While dietary indices derived from dietary intake measures are useful for assessing dietary quality differences between population groups, there is a loss of granularity when dietary data is further reduced into a single dietary quality score, and it can be difficult to identify component dietary determinants and translate these into specific recommendations to inform policy and practice. Additionally, due to the ethnic uniformity of our population, generalisability is limited as it is not a nationally representative sample. Our study also has several strengths, using multiple dietary indices with nested structures provides a broad investigative lens with which to examine dietary patterns. Our sample size and magnitude of complete data are also a strength of this study. Additionally, the BHAS provided a stable population suitable for epidemiological research due to the low migration rates in and out of the community [13]. This study will aid in the interpretation of future studies examining associations between these dietary indices and health outcomes in the BHAS.

## 5. Conclusions

The study showed that this Australian population’s dietary patterns align the closest to the ADG. However, there was a low agreement with international plant-based dietary patterns which have demonstrated several benefits for the environment and population health. While it is still uncertain how transferrable these international plant-based dietary patterns are to an Australian population, further research is warranted into the potential benefits of incorporating elements of these plant-based dietary patterns into the ADG. Our findings also demonstrate that the same food groups in similar dietary indices can exhibit a wide variation in their weighting, highlighting the importance of examining diets from a broad dietary pattern perspective, utilising more than one dietary index.

## Figures and Tables

**Table 1:** Participant characteristics, including demographics, lifestyle factors and self-reported medical conditions, by sex (males/females) and for the cohort as a whole (all).

Characteristics ^1^	All (*n* = 3458)	Males (*n* = 1546)	Females (*n* = 1912)
**Demographics**			
**Age** (**years**)	64 (60–69)	64 (60–69)	64 (59–68)
**Marital Status**			
No partner	18.59 (643)	14.17 (219)	22.18 (424)
Partner	81.41 (2815)	85.83 (1327)	77.82 (1488)
**Ethnicity**			
Caucasian	99.16 (3429)	99.42 (1537)	98.95 (1892)
Other	0.84 (29)	0.58 (9)	1.05 (20)
**Highest level of Education attained**			
School (Primary or Secondary)	48.44 (1675)	49.74 (769)	47.38 (906)
Other educational institution (e.g., TAFE ^3^) college)	31.15 (1077)	30.66 (474)	31.54 (603)
University	19.60 (303)	21.08 (403)	20.44 (706)
**Income (average per annum, before tax)**			
≤$40,000	22.09 (764)	18.24 (282)	25.21 (482)
$40,001 to $80,000	27.82 (962)	29.37 (454)	26.57 (508)
>$80,001	30.88 (1068)	36.35 (562)	26.46 (506)
Prefer not to say	19.20 (664)	16.04 (248)	21.76 (416)
**Lifestyle**			
**Vigorous to moderate physical exercise** (**hours per week**)	8.0 (1.5–18.0)	10.5 (3.0–24.0)	6.0 (0.5–14.0)
**Smoking status**			
Never	49.05 (1696)	44.05 (681)	53.09 (1015)
Previous (ex)	46.24 (1599)	50.91 (787)	42.47 (812)
Current	4.71 (163)	5.05 (78)	4.45 (85)
**Self-Reported Medical Conditions**			
**Cancer**	7.89 (273)	8.80 (136)	7.17 (137)
**High blood pressure** (**ever**)	31.90 (1103)	34.67 (536)	29.65 (567)
**High blood pressure** (**current**)	25.94 (897)	27.75 (429)	24.48 (468)
**High cholesterol** (**ever**)	31.69 (1096)	32.92 (509)	30.7 (587)
**High cholesterol** (**current**)	24.26 (839)	23.74 (367)	24.69 (472)
**Diabetes**	7.72 (267)	9.18 (142)	6.54 (125)
**Kidney Disease**	1.85 (64)	1.62 (25)	2.04 (39)
**COPD ^4^**	1.27 (44)	1.16 (18)	1.36 (26)
**Cardiovascular Disease ^2^**	7.1 (245)	9.4 (146)	5.2 (99)

^1^ Continuous variables (age, vigorous to moderate physical exercise (hours per week)) represented as median (interquartile range); other variables as percentages (numbers) per variable. ^2^ Cardiovascular disease; incidence (ever) of any of the following: angina, claudication, myocardial infarction (MIA), transient ischemic attack (TIA), stroke, carotid surgery, coronary angiogram or coronary bypass. ^3^ TAFE (Technical and Further Education); vocational education and training institution in Australia. ^4^ COPD (Chronic obstructive pulmonary disease).

**Table 2:** Percentage and counts of participants achieving full points for each food group across four dietary indices.

Food Group ^3^	DGI 2013 ^2^	EAT-Lancet Index ^2^	Mediterranean Diet Index	Literature-Based Mediterranean Diet Index ^2^
	% (*n*) ^1^	% (*n*) ^1^	% (*n*) ^1^	% (*n*) ^1^
Fruit ^4^	66.9 (2312)	55.1 (1907)	50.1 (1733)	34.0 (1173)
Vegetables ^4^	49.8 (1723)	64.0 (2214)	49.8 (1723)	85.7 (2963)
Potato	NA ^6^	27.3 (881)	NA ^6^	NA ^6^
Cereals	4.7 (162)	NA ^5^	50.1 (1732)	16.5 (570)
Wholegrains ^4^	60.7 (2098)	1.8 (64)	NA ^6^	NA ^6^
Legumes	NA ^6^	5.1 (175)	51.4 (1779)	27.3 (943)
Nuts	NA ^6^	1.4 (47)	NA ^6^	NA ^6^
Meat ^4^	87.6 (3030)	4.7 (162)	49.4 (1710)	17.6 (609)
Lean Meat Ratio	3.4 (118)	NA ^6^	NA ^6^	NA ^6^
Poultry	NA ^5^	85.9 (2969)	NA ^5^	NA ^5^
Fish	NA ^5^	50.6 (1751)	49.5 (1712)	38.8 (1342)
Egg	NA ^5^	32.1 (1109)	NA ^6^	NA ^6^
Dairy^4^	32.3 (1043)	36.4 (1257)	49.2 (1702)	29.4 (1018)
Low Fat Dairy Ratio	44.2 (1530)	NA ^6^	NA ^6^	NA ^6^
Saturated Fats	61.6 (2132)	NA ^5^	NA ^6^	NA ^6^
Lard	NA ^5^	98.4 (3404)	NA ^6^	NA ^6^
Unsaturated Fats ^4^	4.6 (158)	0.2 (6)	NA ^5^	NA ^5^
Olive Oil	NA ^5^	NA ^5^	21.1 (729)	1.2 (43)
Alcohol	73.5 (2543)	NA ^6^	46.2 (1598)	22.1 (765)
Added Sugar ^4^	67.9 (2349)	55.2 (1909)	NA ^6^	NA ^6^
Added Salt	34.3 (1185)	NA ^6^	NA ^6^	NA ^6^
Discretionary Foods	15.1 (521)	NA ^6^	NA ^6^	NA ^6^
Variety	0.0 (1)	NA ^6^	NA ^6^	NA ^6^
Beverages	49.7 (1720)	NA ^6^	NA ^6^	NA ^6^
Water Ratio	41.6 (1439)	NA ^6^	NA ^6^	NA ^6^

^1^ Percentages (*n*) of participants who achieved the highest score for each food group. ^2^ The EAT-Lancet Index, Dietary Guideline Index (DGI) 2013 and Literature-Based Mediterranean Diet Index (Lit-MedDiet) do not have binary subcomponent scoring systems; for the full details of relative adherence to across the components, please see Tables S1–S8. Positively scored for higher intakes (highlighted in blue); Positively scored for lower intakes (highlighted in pink); Positively scored for moderate intakes (highlighted in grey). ^3^ All food groups that are included in the EAT-Lancet Index, DGI2013, MedDiet, and Lit-MedDiet. ^4^ Food group does not consist of the same dietary variables across all dietary indices, for examples of food and ingredients included in each food group, please see Table S13. ^5^ Food group is not in its own category but is included as a part of another category. ^6^ Food group is not a component of the dietary index.

**Table 3:** Model two beta coefficient estimates of the EAT-Lancet Index, Dietary Guideline Index 2013 (DGI-2013), Mediterranean Diet Index (MedDiet) and the Literature-Based Mediterranean Diet Index (Lit-MedDiet), adjusted for Demographic and Lifestyle Factors.

Predictor ^5^	DGI 2013		EAT-Lancet Index		MedDiet		Lit-MedDiet	
	β (95% CI) ^1,4^	*p* Value	β (95% CI) ^1,4^	*p* Value	β (95% CI) ^1,4^	*p* Value	β (95% CI) ^1,4^	*p* Value
Demographic								
Age (years) ^2,4^	0.01 (0.09, 0.03)	0.001	0.00 (0.06, −0.01)	0.49	0.02 (0.13, 0.05)	0.001	0.02 (0.12, 0.05)	<0.001
Sex		<0.001		<0.001		0.50		<0.001
Male (ref)	ref		ref		ref		ref	
Female	0.73 (0.66, 0.79)		0.36 (0.29, 0.43)		0.04 (−0.03, 0.11)		0.25 (0.18, 0.32)	
Marital Status		0.005		0.54		0.01		0.29
No partner (ref)	ref		ref		ref		ref	
Partner	0.21 (0.13, 0.30)		−0.01 (−0.09, 0.08)		0.08 (−0.01, 0.17)		0.07 (−0.02, 0.16)	
Ethnicity		0.34		<0.001		0.12		0.003
Caucasian (ref)	ref		ref		ref		ref	
Other	−0.21 (−0.55, 0.12)		0.67 (0.32, 1.03)		0.29 (−0.07, 0.65)		0.53 (0.17, 0.89)	
Highest level of Education attained		<0.001		<0.001		0.001		<0.001
School (Primary and Secondary) (ref)	ref		ref		ref		ref	
Other educational institution (e.g., TAFE ^6^)	0.17 (0.10, 0.24)		0.12 (0.05, 0.20)		0.07 (0.00, 0.15)		0.11 (0.32, 0.18)	
University	0.26 (0.18, 0.35)		0.30 (0.21, 0.39)		0.15 (0.06, 0.24)		0.17 (0.08, 0.26)	
Income (average per annum, before tax)		0.11		0.04		0.02		0.19
≤$40,000 (ref)	ref		ref		ref		ref	
$40,001 to $80,000	−0.08 (−0.17, 0.01)		−0.04 (−0.14, −0.06)		0.03 (−0.07, 0.12)		−0.05 (−0.14, 0.05)	
> $80,001	−0.09 (−0.20, 0.00)		0.06 (−0.04, 0.17)		0.13 (0.03, 0.24)		0.03 (−0.07, 0.14)	
Prefer not to say	−0.12 (−0.22, −0.03)		−0.07 (−0.17, 0.03)		−0.01 (−0.12, 0.09)		−0.06 (−0.16, 0.05)	
Lifestyle								
Vigorous/moderate physical activity (hours per week) ^3,4,7^	0.12 (0.08, 0.27)	0.03	0.09 (0.14, 0.33)	<0.001	0.11 (0.20, 0.40)	<0.001	0.15 (0.15, 0.34)	<0.001
Smoking status		<0.001		<0.001		0.12		0.01
Never (ref)	ref		ref		ref		ref	
Former	−0.19 (−0.26, −0.13)		−0.05 (−0.12, 0.01)		0.04 (−0.02, 0.11)		−0.04 (−0.11, 0.03)	
Current	−0.48 (−0.63, −0.33)		−0.41 (−0.57, −0.25)		−0.11 (−0.27, 0.05)		−0.24 (−0.40, −0.08)	

1 β estimates (95% CI) for z-scores of the Dietary Guideline Index 2013 (DGI-2013), EAT-Lancet Index, the Mediterranean Diet Index (MedDiet) and the Literature-Based Mediterranean Diet Index (Lit-MedDiet) based on linear regression modelling. 2 Likelihood ratio tests for non-linearity of age were >0.05 for all dietary indices, therefore age was modelled as a linear term. 3 Likelihood ratio tests for non-linearity for hours of vigorous to moderate physical exercise per week were <0.05 for all dietary indices, therefore fitted with restricted cubic splines. 4 For age, hours of vigorous to moderate physical exercise per week; the difference between the estimate of the mean and (mean–0.5SD to mean + 0.5SD). For all other predictors, the mean difference to the estimate for the reference level (reference level indicated by “ref”). 5 Analyses adjusted for age, sex, marital status, ethnicity, the highest level of education attained, income, hours of vigorous to moderate physical exercise per week, and smoking status (model 2). 6 TAFE (Technical and Further Education); vocational education. 7 Vigorous/moderate physical activity (hours per week); the sum of (hours/week of moderate-intensity physical activities) + 2 × (hours/week of vigorous-intensity physical activities).

**Table 4:** Model three beta coefficient estimates of the EAT-Lancet Index, Dietary Guideline Index 2013 (DGI-2013), Mediterranean Diet Index (MedDiet) and the Literature-Based Mediterranean Diet Index.

Predictor ^3^	DGI 2013		EAT-Lancet Index		MedDiet		Lit-MedDiet	
	β (95% CI) ^1 2^	*p* Value	β (95% CI) ^1,2^	*p* Value	β (95% CI) ^1,2^	*p* Value	β (95% CI) ^1,2^	*p* Value
Cancer	−0.08 (−0.19, 0.03)	0.16	0.00 (−0.12, 0.12)	0.94	−0.11 (−0.24, 0.01)	0.12	−0.07 (−0.19, 0.05)	0.26
High blood pressure (ever)	−0.03 (−0.10, 0.03)	0.33	−0.08 (−0.15, −0.01)	0.02	−0.08 (−0.15, 0.00)	0.04	−0.09 (−0.17, −0.02)	0.01
High blood pressure (current)	−0.04 (−0.11, 0.04)	0.33	−0.11 (−0.18, −0.03)	0.004	−0.10 (−0.17, −0.02)	0.01	−0.10 (−0.17, −0.02)	0.01
High cholesterol (ever)	0.02 (−0.05, 0.08)	0.64	−0.03 (−0.10, 0.05)	0.48	0.01 (−0.06, 0.09)	0.70	0.00 (−0.07, 0.07)	0.96
High cholesterol (current)	−0.01 (−0.08, 0.06)	0.77	−0.05 (−0.13, 0.02)	0.18	−0.02 (−0.10, 0.04)	0.56	−0.03 (−0.11, 0.05)	0.46
Diabetes	0.16 (0.05, 0.28)	0.005	−0.10 (−0.22, −0.02)	0.10	−0.24 (−0.36, −0.11)	<0.001	−0.15 (−0.28, −0.03)	0.02
Kidney Disease	0.16 (−0.07, 0.39)	0.16	0.17 (−0.07, 0.41)	0.16	0.09 (−0.15, 0.33)	0.47	0.15 (−0.09, 0.40)	0.22
COPD ^5^	−0.06 (−0.33, 0.21)	0.67	−0.04 (−0.33, 0.24)	0.76	0.00 (−0.30, 0.29)	0.98	0.00 (−0.30, 0.29)	0.99
Cardiovascular Disease ^4^	0.10 (−0.02, 0.22)	0.09	−0.10 (−0.23, 0.03)	0.12	−0.03 (−0.16, 0.10)	0.61	0.04 (−0.09, 0.16)	0.60

^1^ β estimates (95% CI) for z-scores of the Dietary Guideline Index 2013 (DGI-2013), EAT-Lancet Index, the Mediterranean Diet Index (MedDiet) and the Literature-Based Mediterranean Diet Index (Lit-MedDiet) based on linear regression modelling. ^2^ Mean difference to the estimate for the reference level (reference levels not shown = absence of self-reported medical conditions). ^3^ Analyses adjusted for all the variables in model 2 (age, sex, marital status, ethnicity, highest level of education attained, income, hours of vigorous to moderate physical exercise per week, and smoking status) plus self-reported medical conditions one at a time. Model three was replicated for each medical condition of interest (cancer, high blood pressure (ever/current), high cholesterol (ever/current), diabetes, kidney disease, chronic obstructive pulmonary disease (COPD), cardiovascular disease). ^4^ Cardiovascular disease; incidence (ever) of any of the following: angina, claudication, myocardial infarction (MIA), transient ischemic attack (TIA), stroke, carotid surgery, coronary angiogram, or coronary bypass. ^5^ COPD (Chronic obstructive pulmonary disease).

## Data Availability

The study uses de-identified participant data from the Busselton Population Medical Research Institute (BPMRI) database. Investigators may access BPMRI data if they apply to the Institute for the purpose of original scientific research (email address: busseltonhealthstudy@bpmri.org.au). Applications would be reviewed by the BPMRI Scientific Committee. All data relevant to this study are included in the article.

## Supplementary Materials

The following supporting information can be downloaded at: https://www.mdpi.com/article/10.3390/nu15030659/s1, Table S1 [6]: Dietary Guideline Index 2013 (DGI-2013) composition by dietary guideline component and age group and sex; Table S2 [7,15]: EAT-Lancet Score composition by dietary subcomponent; Table S3 [17]: Mediterranean Diet Index (MedDiet) composition by dietary component; Table S4 [10]: Literature-Based Mediterranean Diet Index (Lit- MedDiet) composition by dietary component; Table S5: Median and (Q1–Q3) of Mediterranean Diet Index subcomponent scores; Table S6: Percent achieving Mediterranean Diet Index subcomponent scores; Table S7: Median and (Q1–Q3) of Literature-Based Mediterranean Diet Index (Lit-MedDiet) subcomponent scores; Table S8: Percent achieving Literature-Based Mediterranean Diet Index (Lit-MedDiet) subcomponent scores; Table S9: Median and (Q1–Q3) of EAT-Lancet Index subcomponent scores; Table S10: Percent achieving EAT-Lancet Index subcomponent scores; Table S11: Median and (Q1–Q3) of DGI-2013 subcomponent scores; Table S12: Percent achieving DGI-2013 subcomponent scores; Table S13: Examples of food and ingredients included in food groups for the EAT-Lancet Index, Dietary Guideline Index 2013 (DGI-2013), the Mediterranean Diet Index and the Literature-Based Mediterranean Diet Index; Table S14 [43]: Model one beta coefficient estimates of the EAT-Lancet Index, Dietary Guideline Index 2013 (DGI-2013), the Mediterranean Diet Index (MedDiet) and the Literature-Based Mediterranean Diet Index (Lit-MedDiet), adjusted for Demographic and Lifestyle Factors.
