# Supplementary material for: Comparison of Four Dietary Pattern Indices in Australian Baby Boomers: Findings from the Busselton Healthy Ageing Study
Source: Nutrients. 2023 Jan 28;15(3):659. doi: 10.3390/nu15030659 (PMC9922020; doi:10.3390/nu15030659)
Supplement: Supplementary file 1 [file nutrients-15-00659-s001.zip › nutrients-2103573-supplementary.pdf]

Supplementary material

Table S1: Dietary Guideline Index 2013 (DGI-2013) composition by dietary guideline component and age group and sex.

Dietary Guideline	Indicator and Description	Score	Criteria for Score[1]			
			**Men 51-70 Years**	**Men 70+ Years**	**Women 51-70 Years**	**Females 70+ Years**
1. Enjoy a wide variety of nutritious foods	Food variety: proportion of food from each of the five core food groups eaten at least one serve per week[3]	0	0-19%	0-19%	0-19%	0-19%
		2	20-39%	20-39%	20-39%	20-39%
		4	40-59%	40-59%	40-59%	40-59%
		6	60-79%	60-79%	60-79%	60-79%
		8	80-91%	80-91%	80-91%	80-91%
		10	91-100%	91-100%	91-100%	91-100%
2. Plenty of vegetables	Total vegetable intake: servings of vegetables per day	0	<1.1 servings	<1 servings	<1 servings	<1 servings
		2	1.1 to <2.2 servings	1 to <2 serving	1 to <2 serving	1 to <2 serving
		4	2.2 to <3.3 servings	2 to <3 servings	2 to <3 servings	2 to <3 servings
		6	3.3 to <4.4 servings	3 to <4 servings	3 to <4 servings	3 to <4 servings
		8	4.4 to <5.5 servings	4 to <5 servings	4 to <5 servings	4 to <5 servings
		10	>=5.5 servings	>=5 servings	>=5 servings	>=5 servings
3. Fruit	Total fruit intake: servings of fruit per day	0	<1 serving	<1 serving	<1 serving	<1 serving
		5	1 to <2 servings	1 to <2 servings	1 to <2 servings	1 to <2 servings
		10	>=2 servings	>=2 servings	>=2 servings	>=2 servings
4. Grain (cereal) foods	Servings of total cereal intake per day	0	0 to <1.2 servings	1 to <0.9 servings	0 to <0.8 servings	0 to <0.5 servings
		1	1.2 to <2.4 serving	0.9 to <1.8 serving	0.8 to <1.6 serving	0.5 to <1 serving
		2	2.4 to <3.6 servings	1.8 to <2.7 servings	1 .6 to <2.4 servings	1 to <2 servings
		3	3.6 to <4.8 servings	2.7 to <3.6 servings	2.4 to <3.2 servings	2 to <3 servings
		4	4.8 to <6 servings	3.6 to <4.5 servings	3.2 to <4 servings	3 to <4 servings
		5	>=6 servings	>=4.5 servings	>=4 servings	>=4 servings
	Mostly wholegrain or high fiber cereals: Type of bread usually consumed	0	White bread	White bread	White bread	White bread
		5	Wholemeal bread	Wholemeal bread	Wholemeal bread	Wholemeal bread
5. Lean meat and poultry, fish, eggs, nuts and seeds, and legumes/beans	Total meat and alternative: servings per day	0	<2.5 servings	<2.5 servings	<2 servings	<2 servings
		5	≥2.5 servings	≥2.5 servings	≥2 servings	≥2 servings
	Lean meat: proportion of lean meats and alternatives to total meat and alternatives per day	0	0-19%	0-19%	0-19%	0-19%
		1	20-39%	20-39%	20-39%	20-39%
		2	40-59%	40-59%	40-59%	40-59%
		3	60-79%	60-79%	60-79%	60-79%
		4	80-91%	80-91%	80-91%	80-91%
		5	91-100%	91-100%	91-100%	91-100%
6. Milk, yoghurt, cheese and/or their alternatives	Total dairy and alternative: servings per day	0	0 to <0.5 servings	0 to <0.7 servings	0 to <0.8 servings	0 to <0.8 servings
		2	0.5 to <1 servings	0.7 to <1.4 servings	0.8 to <1 .6 servings	0.8 to <1 .6 servings
		4	1 to <1.5 serving	1 .4 to <2.1 serving	1.6 to <2.4 serving	1.6 to <2.4 serving
		6	1.5 to <2 servings	2.1 to <2.8 servings	2.4 to <3.2 servings	2.4 to <3.2 servings
		8	2 to <2.5 servings	2.8 to <3.5 servings	3.2 to <4 servings	3.2 to <4 servings
		10	>=2.5 servings	>=3.5 servings	>=4 servings	>=4 servings
	Proportion of low fat diary to total dairy >=50% per day.	0	<50%	<50%	<50%	<50%
		5	>=50%	>=50%	>=50%	>=50%
7. Drink plenty of water	Total beverage intake: servings per day	0	<10 servings	<10 servings	<8 servings	<8 servings
		5	≥10 servings	≥10 servings	≥8 servings	≥8 servings
	Proportion of water to total beverage intake per day	0	<50%	<50%	<50%	<50%
		5	>=50%	>=50%	>=50%	>=50%
8. Limit intake of foods or drinks high in saturated fat,	Limit discretionary food intake: servings per day[2]	0	>=2.5 servings	>=2.5 servings	>=2.5 servings	>=2 servings
		5	1 to <2.5 servings	1 to <2.5 servings	1 to <2.5 servings	1 to <2 servings

and containing added salt and sugar		10	<1 serving	<1 serving	<1 serving	<1 serving
9. Limit intake of foods high in saturated fat	Trim meat: visible fat on meat consumption	0	Eat most of it	Eat most of it	Eat most of it	Eat most of it
		2.5	Eat some of it	Eat some of it	Eat some of it	Eat some of it
		5	Eat as little as possible or Don't eat meat	Eat as little as possible or Don't eat meat	Eat as little as possible or Don't eat meat	Eat as little as possible or Don't eat meat
	Proportion of low fat diary to total dairy >=50% per day.	0	<50%	<50%	<50%	<50%
		5	>=50%	>=50%	>=50%	>=50%
10. Small allowance of unsaturated oils, fats or spreads	Unsaturated spreads and oils: servings per day	0	>4 servings	>4 servings	>2 servings	>2 servings
		10	<=4 servings	<=4 servings	<=2 servings	<=2 servings
11. Limit intake of foods and drinks containing added salt	Salt added to cooking or food	10	no	no	no	no
		0	yes	yes	yes	yes
12. Limit intake of foods and drinks containing added sugars	Limit extra sugar: added to food teaspoons (tsp) per day	0	sugar (tsp) >4	sugar (tsp) >4	sugar (tsp) >4	sugar (tsp) >4
		2	3<sugar (tsp) <=4	3<sugar (tsp) <=4	3<sugar (tsp) <=4	3<sugar (tsp) <=4
		4	2<sugar (tsp) <=3	2<sugar (tsp) <=3	2<sugar (tsp) <=3	2<sugar (tsp) <=3
		6	1<sugar (tsp) <=2	1<sugar (tsp) <=2	1<sugar (tsp) <=2	1<sugar (tsp) <=2
		8	0< sugar (tsp) <=1	0< sugar (tsp) <=0	0< sugar (tsp) <=0	0< sugar (tsp) <=0
		10	sugar (tsp)= 0	sugar (tsp)= 0	sugar (tsp)= 0	sugar (tsp)= 0
13. If you choose to drink alcohol, limit intake	Limit alcohol: servings per day	0	>2 servings	>2 servings	>2 servings	>2 servings
		10	<= 2 servings	<= 2 servings	<= 2 servings	<= 2 servings
	Min total Score	0				
	Max total Score	130				

[1]Criteria of maximum and minimum scores based on the DGI-2013 [1], with criteria added to create intermediate scores. [2]Alcohol excluded, alcohol intake is already captured separately in component 13 instead. [3]Meat and meat products excluded from denominator of ratio for those following vegetarian diet.

Table S2: EAT- Lancet Score composition by dietary subcomponent.

**Dietary Components**	**Score**	**Score criteria[1] (All ages and sex)**
1. Vegetables	0	<100 g/day
	1	100 - 200 g/day
	2	200 - 300 g/day
	3	>300 g/day
2. Fruits	0	<50 g/day
	1	50 - 100 g/day
	2	100 - 200 g/day
	3	>200 g/day
3. Unsaturated Oils[3]	0	<10 g/day
	0.5	10 - 20 g/day
	1	20 - 40 g/day
	1.5	>40 g/day
4. Legumes	0	<18.75 g/day
	1	18.75 - 37.5 g/day
	2	37.5 - 75 g/day
	3	>75 g/day
5. Nuts	0	<12.5 g/day
	1	12.5 - 25 g/day
	2	25 - 50 g/day
	3	>50 g/day
6. Wholegrains	0	<58 g/day
	1	58 - 116 g/day
	2	116 - 232 g/day
	3	>232 g/day
7. Fish	0	<7 g/day
	1	7 - 14 g/day
	2	14 - 28 g/day
	3	>28 g/day
8. Beef/Lamb/Pork[4]	0	> 56 g/day
	2	28 - 56 g/day
	4	14 - 28 g/day
	6	<14 g/day
9. Poultry	0	> 116 g/day
	1	58 - 116 g/day
	2	29 - 58 g/day
	3	<29 g/day
10. Eggs	0	> 50 g/day
	1	25 - 50 g/day
	2	13 - 25 g/day
	3	<13 g/day
11. Dairy	0	> 1000 g/day
	1	500 - 1000 g/day
	2	250 - 500 g/day
	3	<250 g/day
12. Potatoes	0	> 200 g/day
	1	100 - 200 g/day
	2	50 - 100 g/day
	3	<50 g/day

13. Added Sugar	0	> 124 g/day
	1	62 - 124 g/day
	2	31 - 62 g/day
	3	<31 g/day
14. Lard[2]	0	> 10 g/day
	0.5	5 - 10 g/day
	1	1 - 5 g/day
	1.5	0 g/day
Min total Score	0	
Max total Score	42	

[1] Criteria for scores based on the revised EAT-Lancet Index [2]. [2] Lard included as original EAT-Lancet Index recommended [3]. [3] Points allocated to unsaturated oils halved to accommodate for lard inclusion. [4] Beef/lamb/pork combined into one category as the food frequency questionnaire did not distinguish between these meat types.

Table S3: Mediterranean Diet Index (MedDiet) composition by dietary component.

Dietary Components	Score	Score criteria for females (intake g/day)	Score criteria for males (intake g/day)
1. Vegetables	0	below the median	below the median
	1	above the median	above the median
2. Fruit and nuts	0	below the median	below the median
	1	above the median	above the median
3.Cereals	0	below the median	below the median
	1	above the median	above the median
4. Legumes	0	below the median	below the median
	1	above the median	above the median
5.Fish	0	below the median	below the median
	1	above the median	above the median
6. Meat	0	above the median	above the median
	1	below the median	below the median
7. Diary	0	above the median	above the median
	1	below the median	below the median
8. Olive Oil [2]	0	below the median	below the median
	1	above the median	above the median
9. Alcohol	0	5 ≥ ethanol g/day ≥ 25	10 ≥ ethanol g/day ≥ 50
	1	5≤ ethanol g/day≤25	10≤ ethanol g/day≤50
Min total Score	0		
Max total Score	9		

[1] Criteria for scores based on the Mediterranean Diet Index (MedDiet). [2] Olive oil used instead of the saturated fat ratio [4].

Table S4: Literature Based Mediterranean Diet Index (Lit- MedDiet) composition by dietary component.

Dietary Components	Score	Score criteria (All ages and sex)[1]
1. Fruit	0	<150 g/day
	1	150 - 225 g/day
	2	>300 g/day
2. Vegetables	0	<100 g/day
	1	100-250 g/day
	2	>250 g/day
3. Legumes	0	<70 g/week
	1	70-140 g/week
	2	>140 g/week
4. Cereals	0	<130g/day
	1	130-195 g/day
	2	>195 g/day
5. Fish	0	<100 g/week
	1	100-250 g/week
	2	>250 g/week
6. Meat	0	> 120g/day
	1	80-120 g/day
	2	<80 g/day
7. Dairy Products	0	>270g/day
	1	180-270 g/day
	2	<180 g/day
8. Alcohol	0	> 24g/day
	1	<12 g/day
	2	12-24 g/day
9. Olive Oil[2]	0	less than once per week
	1	1 – 6 times per week
	2	daily or 2+ times per day
Min total Score	0	
Max total Score	18	

[1] Criteria for scores based on the Literature-Based Mediterranean Diet Index [5]. [2] Olive oil criteria modified to align with food frequency questionnaire.

Table S5: Median and (Q1-Q3) of Mediterranean Diet Index subcomponent scores.

MedDiet (0-9)	Median (Q1-Q3)[1]
**Total Score**	4 (3-5)
**Food Groups**	
Fruit	1 (0-1)
Vegetables	0 (0-1)
Legumes	1 (0-1)
Cereals	1 (0-1)
Fish	0 (0-1)
Meat	0 (0-1)
Dairy	0 (0-1)
Alcohol	0 (0-1)
Olive Oil	0 (0-0)

[1] Median [Q1-Q3] total scores and subcomponent scores for individual food groups for the Mediterranean Diet Index.

Table S6: Percent achieving Mediterranean Diet Index subcomponent scores.

MedDiet Food Groups	0 points % (n)[1]	1 point % (n)[1]
Fruit	59.9 (1725)	50.1 (1733)
Vegetables	50.2 (1735)	49.8 (1723)
Legumes	48.6 (1679)	51.4 (1779)
Cereals	49.9 (1726)	50.1 (1732)
Fish	50.5 (1746)	49.5 (1712)
Meat	50.6 (1748)	49.4 (1710)
Dairy	50.8 (1756)	49.2 (1702)
Alcohol	53.8 (1860)	46.2 (1598)
Olive Oil	78.9 (2729)	21.1 (729)

[1] Percent (n) of participants achieving minimum and maximum scores for individual food groups of the Mediterranean Diet Index.

Table S7: Median and (Q1-Q3) of Literature-Based Mediterranean Diet Index (Lit-MedDiet) subcomponent scores.

Lit-MedDiet (0-18)	Median (Q1-Q3)[1]
**Total Score**	8 (6-9)
**Food Groups**	
Fruit	1 (0-2)
Vegetables	2 (2-2)
Legumes	0 (0-2)
Cereals	0 (0-1)
Fish	1 (1-2)
Meat	0 (0-1)
Dairy	0 (0-1)
Alcohol	1 (1-1)
Olive Oil	0 (0-0)

[1] Median [Q1-Q3] total scores and subcomponent scores for individual food groups for the Literature-Based Mediterranean Diet Index.

Table S8: Percent achieving Literature-Based Mediterranean Diet Index (Lit-MedDiet) subcomponent scores.

Lit-MedDiet Food Groups	0 points	1 point	2 points
	% (n)[1]	% (n)[1]	% (n)[1]
Fruit	31.8 (1101)	34.2 (1184)	34.0 (1173)
Vegetables	1.2 (43)	13.1 (452)	85.7 (2963)
Legumes	50.4 (1744)	22.3 (771)	27.3 (943)
Cereals	62.9 (2175)	20.6 (713)	16.5 (570)
Fish	22.9 (793)	38.3 (1323)	38.8 (1342)
Meat	63.8 (2205)	18.6 (644)	17.6 (609)
Dairy	60.3 (2085)	29.4 (1018)	10.3 (355)
Alcohol	23.1 (797)	54.8 (1896)	22.1 (765)
Olive Oil	78.4 (2712)	20.3 (703)	1.2 (43)

[1] Percent (n) of participants achieving minimum, intermediate, and maximum scores for individual food groups of the Literature-Based Mediterranean Diet Index.

Table S9: Median and (Q1-Q3) of EAT-Lancet Index subcomponent scores.

EAT-Lancet Index (0- 42)	Median (Q1-Q3)[1]
**Total Score**	19 (17-21)
**Food Groups**	
Fruit	2 (2-3)
Vegetables	3 (2-3)
Legumes	0 (0-1)
Wholegrains	0 (0-1)
Fish	3 (2-3)
Meat	0 (0-0)
Dairy	2 (2-3)
Added Sugar	3 (2-3)
Egg	2 (1-3)
Lard	3 (3-3)
Unsaturated Fat	1 (0-1)
Poultry	3 (3-3)
Potato	2 (1-3)
Nuts	0 (0-0)

[1] Median [Q1-Q3] total scores and subcomponent scores for individual food groups for the EAT-Lancet Index.

Table S10: Percent achieving EAT-Lancet Index subcomponent scores.

EAT-Lancet Index Food Groups	0 points % (n)[1]	1 point % (n)[1]	2 points % (n)[1]	3 points % (n)[1]
Fruit	2.95 (102)	10.6 (367)	26.0 (898)	55.1 (1907)
Vegetables	3.0 (102)	12.17 (421)	21.0 (721)	64.0 (2214)
Legumes	68.1 (2353)	17.4 (603)	9.5 (327)	5.1 (175)
Wholegrains	56.0 (1938)	26.0 (900)	16.1 (556)	1.8 (64)
Fish	7.1 (247)	12.8 (442)	29.4 (1018)	50.6 (1751)
Dairy	4.4 (151)	19.3 (669)	39.9 (1381)	36.3 (1257)
Added Sugar	1.4 (48)	10.4 (359)	33.0 (1142)	55.2 (1909)
Egg	17.9 (620)	27.4 (946)	22.6 (783)	32.1 (1109)
Poultry	0.4 (14)	1.7 (58)	12.1 (417)	85.9 (2969)
Potato	9.1 (316)	37.3 (1289)	25.9 (895)	27.7 (958)
Nuts	76.2 (2634)	14.8 (513)	7.6 (264)	1.4 (47)
**EAT-Lancet Score Index Groups**	**0 points % (n)**	**2 points % (n)**	**4 points % (n)**	**6 points % (n)**
Meat	86.7 (2998)	6.6 (228)	2.0 (70)	4.7 (162)
**EAT-Lancet Score Index Groups**	**0 points % (n)**	**0.5 points % (n)**	**1 points % (n)**	**1.5 points % (n)**
Lard	0.4 (13)	0.5 (19)	0.6 (22)	98.4 (3404)
Unsaturated Fat	47.3 (1635)	46.4 (1603)	6.2 (214)	0.1 (6)

[1] Percent (n) of participants achieving minimum, intermediate, and maximum scores for individual food groups of the EAT-Lancet Index.

Table S11: Median and (Q1-Q3) of DGI-2013 subcomponent scores.

DGI-2013 Score (0- 130)	Median (Q1-Q3)[1]
**Total Score**	70 (59-81)
**Food Groups**	
Vegetables	8 (6-10)
Fruit	10 (5-10)
Cereals	1 (1-2)
Wholegrain Emphasis	5 (0-5)
Meat	5 (5-5)
Lean Meat Ratio	3 (1-3)
Dairy	6 (4-10)
Low Fat Dairy Ratio	0 (0-5)
Beverages	0 (0-5)
Water Ratio	0 (0-5)
Discretionary Foods	5 (0-5)
Variety	4 (2-4)
Saturated Fats	5 (3-5)
Unsaturated Fats	0 (0-0)
Added Salt	0 (0-10)
Added Sugar	10 (8-10)
Alcohol	10 (0-10)

[1] Median [Q1-Q3] total scores and subcomponent scores for individual food groups for the DGI-2013.

Table S12: Percent achieving DGI-2013 subcomponent scores.

DGI-2013 Score Food Groups	0 points % (n)[1]	1 point % (n)[1]	2 points % (n)[1]	3 points % (n)[1]	4 points % (n)[1]	5 points % (n)[1]
Cereals	21.7 (750)	37.3 (1289)	22.2 (768)	9.1 (316)	5.0 (173)	4.7 (162)
Lean Meat Ratio	9.1 (316)	19.1 (659)	20.9 (723)	37.0 (1278)	10.5 (364)	3.4 (118)
	**0 points % (n)**	**2 points % (n)**	**4 points % (n)**	**6 points % (n)**	**8 points % (n)**	**10 points % (n)**
Vegetables	0.9 (31)	5.9 (203)	10.6 (366)	15.2 (526)	17.6 (609)	49.8 (1723)
Variety	5.0 (173)	42.2 (1458)	46.1 (1593)	6.7 (233)	0.0 (0)	0.0 (1)
Dairy	7.8 (269)	12.4 (429)	18.3 (632)	15.8 (545)	13.5 (466)	32.3 (1117)
Added Sugar	4.1 (142)	3.2 (110)	4.0 (140)	7.8 (269)	13.0 (448)	67.9 (2349)
	**0 points % (n)**	**5 points % (n)**	**10 points % (n)**			
Fruit	14.5 (500)	18.7 (646)	66.9 (2312)			
Discretionary Foods	45.8 (1585)	39.1 (1352)	15.1 (521)			
	**0 points % (n)**	**2.5 points % (n)**	**5 points % (n)**			
Saturated Fats	13.6 (472)	24.7 (854)	61.6 (2132)			
	**0 points % (n)**	**10 points % (n)**				
Added Salt	65.7 (2273)	34.3 (1185)				
Alcohol	26.5 (915)	73.5 (2543)				
	**0 points % (n)**	**5 points % (n)**				
Unsaturated Fats	95.4 (3300)	4.6 (158)				
Water Ratio	58.4 (2019)	41.6 (1439)				
Beverages	50.3 (1738)	49.7 (1720)				
Meat	12.4 (428)	87.6 (3030)				
Low Fat Dairy Ratio	55.8 (1928)	44.3 (1530)				
Wholegrain Emphasis	39.3 (1360)	60.7 (2098)				

[1] Percent (n) of participants achieving minimum, intermediate, and maximum scores for individual food groups of the DGI-2013.

Table S13. Examples of food and ingredients included in food groups for the EAT-Lancet Index , Dietary Guideline Index 2013 (DGI-2013), the Mediterranean Diet Index, and the Literature-Based Mediterranean Diet Index.

Food group	DGI 2013[1]	EAT-Lancet Index [1]	MedDiet [1]	Lit- MedDiet[1]
Fruit	All fruits (fresh and tinned)	All fruits (fresh and tinned)	All fruits (fresh and tinned), All nuts (including peanut spread)	All fruits (fresh and tinned)
Vegetables	All vegetables	All vegetables (except potatoes)	All vegetables	All vegetables
Potato	NA[5]	Potatoes (boiled, mashed, baked)	NA[5]	NA[5]
Cereals	All cereals (rice, bread, oats, corn, pasta, crackers, cereal)	NA[4]	All cereals (rice, bread, oats, corn, pasta, crackers, cereal)	All cereals (rice, bread, oats, corn, pasta, crackers, cereal)
Wholegrains	Wholemeal bread	[c]Whole bread, brown rice, whole oats, bran, wheat flakes	NA[5]	NA[5]
Legumes	NA[4]	Lentils, beans, soybeans (peas excluded [2])	Lentils, beans, soybeans (peas excluded [2])	Lentils, beans, soybeans (peas excluded [2])
Nuts	NA[4]	All nuts (including peanut spread)	NA[4]	NA[5]
Meat	Beef, lamb, pork, poultry (including their processed meats eg. Sausage, sandwich meat), fish, meat alternatives (legumes, egg, nuts)	Beef, lamb, pork, poultry (including their processed meats e.g. Sausage, sandwich meat)	Beef, lamb, pork, poultry (including their processed meats e.g. Sausage, sandwich meat)	Beef, lamb, pork, poultry (including their processed meats e.g. Sausage, sandwich meat)
Lean Meat Ratio	Lean meat (beef, lamb, pork, poultry) and alternatives (legumes, egg, nuts)[3]	NA[5]	NA[5]	NA[5]
Poultry	NA[4]	All poultry	NA[4]	NA[4]
Fish	NA[4]	All fish (fresh and tinned) and seafood (e.g. prawns, crabs)	All fish (fresh and tinned) and seafood (e.g. prawns, crabs)	All fish (fresh and tinned) and seafood (e.g. prawns, crabs)
Egg	NA[4]	Poultry eggs	NA	NA
Dairy	Milk, yogurt, cheese, butter and cream	Milk, yogurt, cheese, butter and cream	Milk, yogurt, cheese, butter and cream	Milk, yogurt, cheese, butter and cream
Low Fat Dairy Ratio	[c]Low-fat milk, yogurt, cheese, butter	NA[5]	NA[5]	NA[5]
Saturated Fats	Visible fat on meat	NA[4]	NA[5]	NA[5]
Lard	NA[4]	Lard	NA[5]	NA[5]
Unsaturated Fats	Unsaturated plant oils and spreads	Unsaturated plant oils	NA[4]	NA[4]
Olive Oil	NA[4]	NA[4]	Olive oil	Olive oil
Alcohol	Ethanol from all alcoholic beverages	NA[5]	Ethanol from all alcoholic beverages	Ethanol from all alcoholic beverages
Added Sugar	All sugar added to food by individual	All sugar included as ingredients in manufactured foods and beverages	NA[5]	NA[5]
Added Salt	Salt added to cooking/food	NA[5]	NA[5]	NA[5]
Discretionary Foods	Sweets, salty snacks, processed meat and high fat processed foods	NA[5]	NA[5]	NA[5]
Variety	Proportion of food from each core group (fruit, vegetables, meat, dairy, cereals)	NA[5]	NA[5]	NA[5]
Beverages	All beverages (water, tea, juices, soda, alcoholic beverages)	NA[5]	NA[5]	NA[5]
Water Ratio	Water[3]	NA[5]	NA[5]	NA[5]

[1]Examples of foods included in each food category for the the EAT-Lancet Index, Dietary Guideline Index (DGI) 2013, Mediterranean Diet Index (MedDiet), and Literature-Based Mediterranean Diet Index (Lit-MedDiet).[2] Peas were included as a vegetable, as peas were grouped with vegetables in the food frequency questionnaire but fresh and dried peas could not be distinguished (fresh peas are classified by US dietary guidelines as vegetable, while dried peas are classified as a legume [6]). [3] Food group examples that form the numerator of a ratio component. Positively scored for higher intakes; Positively scored for lower intakes; Positively scored for moderate intakes. [4] Food group is not in its own category but is included in another category. [5]Food component not part of dietary score.

Table S14. Model one beta coefficient estimates of the EAT-Lancet Index, Dietary Guideline Index 2013 (DGI-2013), the Mediterranean Diet Index (MedDiet), and the Literature-Based Mediterranean Diet Index (Lit-MedDiet), adjusted for Demographic and Lifestyle Factors.

Predictor	DGI-2013		EAT-Lancet Index		MedDiet		Lit-MedDiet	
	β **(95% CI)** [1, 3]	**P value**	***β*** **(95% CI)** [1, 3]	**P value**	***β*** **(95% CI)** [1, 3]	**P value**	***β*** **(95% CI)** [1, 3]	**P value**
**Demographic**								
Age (years) [2, 3]	0.01 (0.1, 0.03)	0.001	0.00 (-0.06, 0.01)	0.49	0.02 (0.13, 0.05)	0.004	0.02 (0.12, 0.05)	<0.001
Sex		<0.001		<0.001		0.67		<0.001
Male (ref)	ref		ref		ref		ref	
Female	0.72 (0.66, 0.78)		0.34 (0.27, 0.41)		0.00 (-0.07, 0.07)		0.21 (0.15, 0.28)	
Marital Status		0.01		0.57		0.01		0.26
No partner (ref)	ref		ref		ref		ref	
Partner	0.24 (0.16, 0.32)		0.02 (-0.07, 0.10)		0.08 (-0.01, 0.17)		0.08 (-0.01, 0.17)	
Ethnicity		0.28		<0.001		0.20		0.01
Caucasian (ref)	ref		ref		ref		ref	
Other	-0.19 (-0.53, 0.14)		0.67 (0.31, 1.03)		0.25 (-0.11, 0.61)		0.51 (0.15, 0.87)	
Highest level of Education attained		<0.001		<0.001		<0.001		0.005
School (ref)	ref		ref		ref		ref	
Other educational institution (e.g. TAFE, college)	0.17 (0.10, 0.24)		0.13 (0.06, 0.21)		0.08 (0.00, 0.16)		0.11 (0.04, 0.19)	
University	0.29 (0.21, 0.38)		0.33 (0.24, 0.42)		0.16 (0.07, 0.25)		0.18 (0.09, 0.27)	
Income (average before per annum)		0.17		0.03		0.01		0.17
<= $40,000 (ref)	ref		ref		ref		ref	
$40,001 to $80,000	-0.07 (-0.17, 0.02)		-0.03 (-0.12, 0.07)		0.04 (-0.06, 0.14)		-0.04 (-0.13, 0.06)	
> $80,001	-0.08 (-0.18, 0.02)		0.08 (-0.02, 0.19)		0.16 (0.05, 0.26)		0.005 (-0.05, 0.16)	
Prefer not to say	-0.11 (-0.21, -0.01)		-0.06 (-0.16, 0.05)		0.00 (-0.11, 0.11)		-0.05 (-0.15, 0.06)	

[1] ***β*** estimates (95% CI) for z-scores of the Dietary Guideline Index 2013 (DGI-2013), EAT-Lancet Index, the Mediterranean Diet Index (MedDiet), and the Literature-Based Mediterranean Diet Index (Lit-MedDiet) based on linear regression modelling. [2] Likelihood ratio tests for non-linearity of age were >0.05 for all dietary indices, therefore age was modelled as a linear term. [3] For age, difference between the estimate of the mean and (mean - 0.5SD to mean + 0.5SD). For all other predictors, mean difference to the estimate for the reference level (reference level indicated by "ref").[4] Analyses adjusted for age, sex, marital status, ethnicity, highest level of education attained, and income (model 1).

References

1. Thorpe, M.G.; Milte, C.M.; Crawford, D.; McNaughton, S.A. A Revised Australian Dietary Guideline Index and Its Association with Key Sociodemographic Factors, Health Behaviors and Body Mass Index in Peri-Retirement Aged Adults. *Nutrients* **2016**, *8*, 160, doi:10.3390/nu8030160.

2. Stubbendorff, A.; Sonestedt, E.; Ramne, S.; Drake, I.; Hallström, E.; Ericson, U. Development of an EAT-Lancet Index and Its Relation to Mortality in a Swedish Population. *Am. J. Clin. Nutr.* **2022**, *115*, 705–716, doi:10.1093/ajcn/nqab369.

3. Willett, W.; Rockström, J.; Loken, B.; Springmann, M.; Lang, T.; Vermeulen, S.; Garnett, T.; Tilman, D.; DeClerck, F.; Wood, A.; et al. Food in the Anthropocene: The EAT– Lancet Commission on Healthy Diets from Sustainable Food Systems. *The Lancet* **2019**, *393*, 447–492, doi:http://dx.doi.org.ezproxy.library.uwa.edu.au/10.1016/S0140-6736(18)31788-4.

4. Hodge, A.M.; Bassett, J.K.; Dugué, P.-A.; Shivappa, N.; Hébert, J.R.; Milne, R.L.; English, D.R.; Giles, G.G. Dietary Inflammatory Index or Mediterranean Diet Score as Risk Factors for Total and Cardiovascular Mortality. *Nutr. Metab. Cardiovasc. Dis. NMCD* **2018**, *28*, 461–469, doi:10.1016/j.numecd.2018.01.010.

5. Sofi, F.; Macchi, C.; Abbate, R.; Gensini, G.F.; Casini, A. Mediterranean Diet and Health Status: An Updated Meta-Analysis and a Proposal for a Literature-Based Adherence Score. *Public Health Nutr.* **2014**, *17*, 2769–2782, doi:10.1017/S1368980013003169.

6. *Dietary Guidelines for Americans, 2020-2025*; U.S. Department of Agriculture and U.S. Department of Health and Human Services, 2020;
